# Inclisiran: a new generation of lipid-lowering siRNA therapeutic

**DOI:** 10.3389/fphar.2023.1260921

**Published:** 2023-10-13

**Authors:** Yanzhen Zhang, Huaigang Chen, Lang Hong, Hong Wang, Bin Li, Mengyin Zhang, Jiamei Li, Liu Yang, Fan Liu

**Affiliations:** ^1^ Department of Rheumatology, Shunyi Hospital, Beijing Traditional Chinese Medicine Hospital, Beijing, China; ^2^ Medical College of Nanchang University, Nanchang, China; ^3^ Department of Cardiology, Jiangxi Provincial People’s Hospital, The First Affiliated Hospital of Nanchang Medical College, Nanchang, China; ^4^ Pharmacy Department, Jiangxi Provincial People’s Hospital, The First Affiliated Hospital of Nanchang Medical College, Nanchang, China; ^5^ Laboratory Department, Jiangxi Provincial People’s Hospital, The First Affiliated Hospital of Nanchang Medical College, Nanchang, China; ^6^ Department of Hematology Jiangxi Hospital of Traditional Chinese Medicine, Nanchang, China

**Keywords:** lipid-lowering therapies, cardiovascular risk, PCSK9, Inclisiran, siRNA

## Abstract

Atherosclerotic heart disease (AHD) is a major cause of morbidity and mortality worldwide. Lowering low-density lipoprotein cholesterol (LDL-C) levels is a key strategy to prevent and treat AHD. Inclisiran is a novel siRNA drug that targets proprotein convertase subtilisin/kexin type 9 (PCSK9) gene expression and reduces LDL-C levels with only two or three injections per year. This review summarizes the mechanism, efficacy, safety, and applications of Inclisiran in various populations and settings, based on recent literature. It also compares Inclisiran with other lipid-lowering drugs, especially other PCSK9 inhibitors. We conclude that Inclisiran is a promising lipid-lowering agent that can provide convenience and effectiveness for patients with high cardiovascular risk. However, some challenges and limitations remain for Inclisiran, such as its long-term safety and efficacy, its cost-effectiveness and accessibility, and its interactions and synergies with other drugs. These issues need further investigation and evaluation in future studies.

## 1 Introduction

Atherosclerotic heart disease (AHD) remains a global public health problem despite of significant advances in its prevention and treatment (Dong et al., 2022). It is caused by the accumulation of cholesterol and other substances in the arterial walls, which led to narrowing and hardening of the arteries and reduce blood flow to the heart and other organs. This can result in chest pain, heart attack, stroke or death. A positive correlation between circulating low-density lipoprotein cholesterol (LDL-C) levels and coronary heart disease has been demonstrated by numerous epidemiological and clinical studies (Mortensen and Nordestgaard, 2020). LDL-C is a kind of cholesterol that can damage the arteries and increase the risk of atherosclerosis (Ference et al., 2013; Liu et al., 2021). LDL-C is the main carrier of cholesterol in the blood and can be taken up by the arterial wall cells via LDL receptors, contributing to plaque formation and inflammation. This process can lead to atherosclerosis and cardiovascular disease (Robinson et al., 2018). In China, the percentage of adults with dyslipidemia, which is an abnormal level of lipids in the blood, is as high as 33.8%, while the percentage of those with increased LDL-C level, which is a major risk factor for coronary artery disease, is 4.0%. These statistics indicate a high prevalence and burden of lipid metabolic disorders in China (Lu et al., 2021). How to effectively reduce LDL-C is one of the research questions to be answered.

In recent years, PCSK9 has gained much attention as a star target for reducing LDL-C levels in patients (German and Shapiro, 2020). PCSK9 is a protein that regulates the degradation of LDL receptors on the surface of liver cells, thereby affecting the clearance of LDL-C from the blood. PCSK9 binds to LDL receptors and promotes their degradation in lysosomes, thus reducing the number of LDL receptors available for clearing LDL-C from the blood. Therefore, inhibiting PCSK9 can increase the expression of LDL receptors and lower LDL-C levels (Tibolla et al., 2011). At present, the drug research and development targeting PCSK9 is mainly focused on preventing the binding of PCSK9 to the low-density lipoprotein (LDL) receptor. There are two main types of drugs that can achieve this goal: monoclonal antibodies and small interfering RNAs (siRNA). Monoclonal antibodies are proteins that can bind to PCSK9 and block its interaction with LDL receptors (Stroes et al., 2014) SiRNA are short nucleic acids that can silence the expression of PCSK9 gene in liver cells (Reyes-Soffer et al., 2017), inhibiting the expression and secretion of PCSK9 (Fitzgerald et al., 2017).

Among these drugs, monoclonal antibodies that block the binding of PCSK9 to LDL receptors are now available in China. The lipid-lowering effect of this drug is very effective, require monthly or every biweekly injection. However, the shortcoming of low compliance and the high cost of preparation of monoclonal antibody preparations are still the major obstacles to the development of these drugs (Kaddoura et al., 2020). This drug refers to the monoclonal antibodies that target PCSK9, such as evolocumab and alirocumab. These drugs can significantly reduce LDL-C levels and cardiovascular events in patients with dyslipidemia (Stroes et al., 2014; Robinson et al., 2015; Sabatine et al., 2017; Diaz et al., 2021). In the meantime, Alirocumab can reduced the risk of any stroke and ischemic stroke without increasing hemorrhagic stroke (Jukema et al., 2019). However, they also have some drawbacks, such as low patient adherence due to frequent injections, high production cost and limited accessibility. Therefore, new lipid-lowering drugs are needed urgently.

Recent studies have shown that PCSK9 is a key protein that regulates low density lipoprotein receptor (LDL-R) expression in hepatocytes, and is positively correlated with LDL-C levels (Tibolla et al., 2011). Targeting PCSK9 has brought new opportunities for lipid-lowering therapies. However, current treatments still face challenges such as poor medication adherence (Jahangir et al., 2021). However, as an emerging therapeutic approach, the long-term safety, cardiovascular risk reduction efficacy, and applicability across diverse populations of siRNA therapeutics need to be verified through more studies (Kosmas et al., 2018).

Inclisiran (trade name Leqvio ^®^) is a lipid-lowering novel drug developed by Novartis Group. It is the first siRNA drug approved for the treatment of hypercholesterolemia or mixed dyslipidemia, as well as ASCVD or heterozygous familial hypercholesterolemia (HeFH) patients who need further lowering of LDL-C levels. It is a short-chain, synthetic small interfering ribonucleic acid (siRNA) (Khvorova, 2017). SiRNAs are types of double-stranded RNAs that can induce gene silencing by degrading the complementary mRNAs in a sequence-specific manner. However, some issues and challenges remain for Inclisiran, such as its effect on type 2 diabetes, its long-term outcomes, its affordability and availability, and its combination with other drugs. Moreover, the molecular mechanisms and pathways of Inclisiran-mediated LDL-C reduction and cardiovascular protection need to be clarified, and the development of novel siRNA drugs for lipid metabolism or cardiovascular disease may offer new opportunities for personalized and precision medicine. Considering the important value of Inclisiran as a novel siRNA drug in lipid-lowering therapy, we reviewed the recent literature on this drug, and outlined its mechanism of action, pharmacodynamics, safety evaluation, application in China and future application, etc., to provide a comprehensive overview of the advantages and existing problems of Inclisiran, and to offer reference for its position in lipid-lowering therapy.

## 2 Methods

### 2.1 Literature search

A literature search was conducted using PubMed, EMBASE, and SinoMed databases. Search terms pre-defined in titles, abstracts, and keywords were used to identify pertinent studies. The retrieval period spanned from the inception of the databases up to March 2023.

## 3 Inclisiran delivery and mechanism of action

### 3.1 Mechanism of action

**FIGURE 1:**
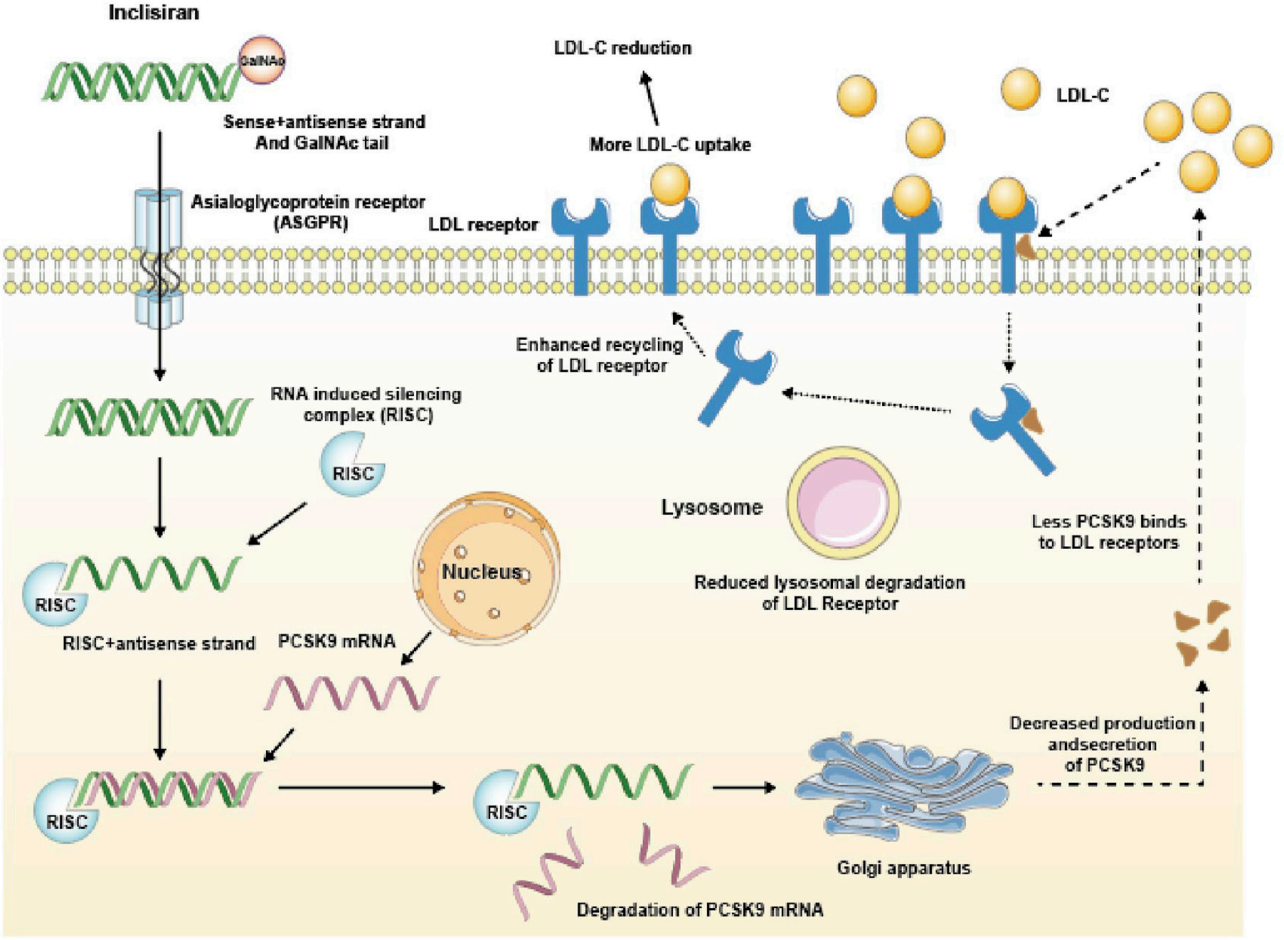
Mechanism of action of Inclisiran. GalNAc tail, N-acetylgalactosamine tail; RISC, RNA-induced silencing complex; PCSK9, Proprotein convertase subtilisin/kexin type 9.

Inclisiran specifically binds to n-acetylgalactosamine (GalNAc) and the asialoglycoprotein receptor (ASGPR) on liver cell membranes (Kosmas et al., 2018). GalNAc is a sugar molecule that enhances the affinity and specificity of Inclisiran for ASGPR, which is a receptor that mediates the endocytosis of glycoproteins in liver cells. After entering the hepatocytes, it binds to the RNA-induced silencing complex (RISC). RISC is a protein complex that recognizes and cleaves the target mRNA based on the guide strand of siRNA. At the same time, it binds to the mRNA who encoding the PCSK9 protein mediated by the antisense chain, which leads to a decrease in PCSK9 protein production. PCSK9 is a protein that regulates the expression of LDL receptors on the surface of liver cells. PCSK9 binds to LDL receptors and promotes their degradation in lysosomes, thus reducing the number of LDL receptors available for clearing LDL-C from the blood. Therefore, by reducing PCSK9 production, Inclisiran can enhance LDL receptor expression and lower LDL-C levels. To illustrate the mechanism of action of Inclisiran, we present a schematic diagram in Figure 1.

### 3.2 Inclisiran efficacy

After a single dose of Inclisiran, the level of LDL-C was decreased by about 50% and maintained for up to 6 months (Barale et al., 2021; Miname et al., 2021). It is noteworthy that the silencing complex remains active after mRNA degradation occurs. Therefore, the lipid-lowering effect of Inclisiran is effective in the long term.

## 4 Clinical efficacy, safety evaluation and exploration and application in China for different types of hypercholesterolemia and cardiovascular disease patients

### 4.1 Clinical trials of inclisiran for hypercholesterolemia

Inclisiran is involved in five global studies: ORION 4, 9, 10, 11, 18. These studies are phase III, double-blind, randomized, placebo-controlled trials that aim to evaluate the efficacy and safety of Inclisiran in patients with different types of hypercholesterolemia.

#### 4.1.1 ORION 9: inclisiran for heterozygous familial hypercholesterolemia

ORION 9 enrolled patients with heterozygous familial hypercholesterolemia (HeFH), a genetic disorder that causes high LDL-C levels and increased risk of cardiovascular disease. The trial tested the hypothesis that Inclisiran, an innovative siRNA agent that silences PCSK9 gene expression in the liver and increases LDL receptor availability, would reduce LDL-C levels more than placebo in patients who were on maximally tolerated statin and ezetimibe.

#### 4.1.2 ORION 10 and 11: inclisiran for atherosclerotic cardiovascular disease or risk equivalents

ORION 10 and 11 enrolled patients with atherosclerotic cardiovascular disease (ASCVD) or ASCVD risk equivalents, such as diabetes mellitus, chronic kidney disease or peripheral artery disease (Ray et al., 2020). These are conditions that can damage the blood vessels and impair the blood flow to the heart and other organs. The patients enrolled in the study received at least 30 days of treatment with a statin at the maximum tolerable dose. Statins are drugs that can lower LDL-C levels by inhibiting the enzyme that produces cholesterol. The dose of statins was constant throughout the study with or without ezetimibe. Ezetimibe is a cholesterol absorption inhibitor that can lower LDL-C levels by blocking the uptake of cholesterol from the intestine. Enrolled patients were randomized to the Inclisiran group or the placebo group. On the basis of the maximum tolerable dose of statin ± ezetimibe, Inclisiran 300 mg or placebo was given subcutaneously at D0, D90, D270, and D450. The primary endpoint of the studies was the percentage change in LDL-C levels from baseline to day 510. The secondary endpoints included the absolute change in LDL-C levels from baseline to day 510, the time-adjusted percentage change in LDL-C levels from day 90 to day 540, and the safety and tolerability of Inclisiran. The Inclisiran group showed a 51% decrease in LDL-C levels, compared to the placebo group, which was statistically significant and clinically meaningful (Leiter et al., 2019; Jahangir et al., 2021). The reduction was consistent and sustained across all subgroups and time points. Inclisiran also reduced other lipid parameters, such as non-HDL-C, apolipoprotein B and lipoprotein (a). Inclisiran was well tolerated and had a similar safety profile to placebo. The most common adverse events were injection site reactions, which were mild or moderate and self-limiting.

#### 4.1.3 ORION-18: inclisiran for asian population

Inclisiran’s phase III clinical study-ORION-18, which is also taking place in China and other Asian countries for the first time. This study will enroll about 1,500 patients with primary hypercholesterolemia or mixed dyslipidemia who are not adequately controlled by statins alone or in combination with other lipid-lowering therapies. The primary endpoint is the percentage change in LDL-C levels from baseline to day 510. This study will provide more evidence for the efficacy and safety of Inclisiran in Asian populations, who may have different genetic and environmental factors that affect their lipid metabolism and response to treatment (Jahangir et al., 2021; Ray et al., 2023).

#### 4.1.4 ORION 4: inclisiran for cardiovascular outcomes

Additionally, ORION4 studies are being conducted to further assess the long-term effectiveness, safety, and cardiovascular benefits of Inclisiran (Brandts and Ray, 2021). This is a large-scale, multicenter, randomized trial that will enroll about 15,000 patients with ASCVD or high risk of ASCVD who have elevated LDL-C levels despite optimal lipid-lowering therapy. It will compare the effects of Inclisiran versus placebo on cardiovascular outcomes. The primary outcome is the composite of coronary heart disease death, non-fatal myocardial infarction, fatal or non-fatal ischemic stroke or coronary revascularization. These are major adverse cardiovascular events (MACE) that can cause significant morbidity and mortality. The trial will follow the patients for at least 4 years and the results could be announced in 2025. This trial will provide important evidence for the long-term benefits and safety of Inclisiran in reducing cardiovascular risk.

A comprehensive summary of the details and findings of the clinical trials of Inclisiran for various hypercholesterolemia populations is given in the table below (See Table 1).

**TABLE 1:** Characteristics and outcomes of the clinical trials of Inclisiran for hypercholesterolemia.

Name	Population	Inclusion criteria	Exclusion criteria	Trial design	Follow-up time	Primary endpoint	Secondary endpoint
ORION-9	Patients with HeFH	18–80 years old, genetic or clinical diagnosis of HeFH, LDL-C ≥ 2.6 mmol/L, maximum tolerated dose of statin ± other lipid-lowering drugs ≥30 days	Pregnant or lactating women, liver dysfunction, other serious diseases or conditions unsuitable for trial participation	Double-blind, randomized, placebo-controlled, multicenter, phase III trial, randomization ratio of 1:1	Observation and measurement on day 0, day 90, day 180, day 270, day 360, day 450 and day 540, for a total of 18 months	Percentage change in LDL-C level from baseline to day 510	Absolute change in LDL-C level from baseline to day 510; time-adjusted percentage change in LDL-C level from day 90 to day 540; safety and tolerability of Inclisiran
ORION-10	Patients with ASCVD or risk equivalent (US)	18–85 years old, with ASCVD or risk equivalent (such as diabetes, chronic kidney disease or peripheral artery disease), LDL-C ≥ 1.8 mmol/L (70 mg/dL), maximum tolerated dose of statin ± other lipid-lowering drugs ≥30 days	Pregnant or lactating women, liver dysfunction, other serious diseases or conditions unsuitable for trial participation	Double-blind, randomized, placebo-controlled, multicenter, phase III trial, randomization ratio of 1:1	Observation and measurement on day 0, day 90, day 180, day 270, day 360, day 450 and day 540, for a total of 18 months	Percentage change in LDL-C level from baseline to day 510	Absolute change in LDL-C level from baseline to day 510; time-adjusted percentage change in LDL-C level from day 90 to day 540; safety and tolerability of Inclisiran
ORION-11	Patients with ASCVD or risk equivalent (Europe)	18–85 years old, with ASCVD or risk equivalent (such as diabetes, chronic kidney disease or peripheral artery disease), LDL-C ≥ 1.8 mmol/L (70 mg/dL), maximum tolerated dose of statin ± other lipid-lowering drugs ≥30 days	Pregnant or lactating women, liver dysfunction, other serious diseases or conditions unsuitable for trial participation	Double-blind, randomized, placebo-controlled, multicenter, phase III trial, randomization ratio of 1:1	Observation and measurement on day 0, day 90, day 180, day 270, day 360, day 450 and day 540, for a total of 18 months	Percentage change in LDL-C level from baseline to day 510	Absolute change in LDL-C level from baseline to day 510; time-adjusted percentage change in LDL-C level from day 90 to day 540; safety and tolerability of Inclisiran
ORION-4	Patients with ASCVD or high risk (global)	55–80 years old, with ASCVD or high risk (such as diabetes, chronic kidney disease or peripheral artery disease), LDL-C ≥ 1.8 mmol/L (70 mg/dL), optimized lipid-lowering therapy ≥30 days	Pregnant or lactating women, liver dysfunction, other serious diseases or conditions unsuitable for trial participation	Randomized, placebo-controlled, multicenter phase III trial to evaluate the effect of Inclisiran on cardiovascular outcomes. Randomization ratio of 1:1	At least 4 years of follow-up, Observe and test every 6 months	Composite endpoint of coronary heart disease death, nonfatal myocardial infarction, fatal or nonfatal ischemic stroke or coronary revascularization (major adverse cardiovascular events, MACE)	Other cardiovascular outcomes and safety and tolerability of Inclisiran
ORION-18	Patients with primary hypercholesterolemia or mixed hyperlipidemia (Asia)	18–85 years old -with primary hypercholesterolemia or mixed hyperlipidemia -LDL-C ≥ −2.6 mmol/L (100 mg/dL) -maximum tolerated dose of statin ± other lipid-lowering drugs ≥30 days	Pregnant or lactating women -liver dysfunction -other serious diseases or conditions unsuitable for trial participation	Double-blind -randomized -placebo-controlled -multicenter phase III trial to evaluate the efficacy and safety of Inclisiran in Asian populations Randomization ratio of −1:1	Observation and measurement on day, 0, day, 90, day, 180, day, 270, day, 360, day, 450 and day, 540, for a total of, 18 months	Percentage change in LDL-C level from baseline to day,510	Other lipid parameters and safety and tolerability of Inclisiran

HeFH, heterozygous familial hypercholesterolemia; LDL-C, Low-Density Lipoprotein Cholesterol; ASCVD, atherosclerotic cardiovascular disease.

### 4.2 Safety of inclisiran

#### 4.2.1 Advantages of inclisiran safety profile

Compared with other lipid-lowering therapies, Inclisiran has a low risk of adverse events and no major safety concerns. The most common adverse event reported in the Inclisiran group was injection site reaction, which occurred in about 5% of the patients. However, these reactions were mostly mild and transient, and none of them were severe or persistent. Injection site reactions may be related to the lipid nanoparticles used for siRNA delivery and may decrease with repeated administration. Moreover, Inclisiran did not cause any significant toxicity in liver, kidney, muscle and platelet functions. Laboratory examination showed that there was no change in the levels of alanine aminotransferase, aspartate aminotransferase, creatinine, creatine kinase or platelet count after Inclisiran treatment. Inclisiran also did not affect the cardiovascular system. There was no difference in blood pressure, heart rate or electrocardiogram parameters between the Inclisiran group and the placebo group. Therefore, Inclisiran appears to be a safe and well-tolerated drug for lowering LDL-C levels in patients with hypercholesterolemia or mixed dyslipidemia (Hardy et al., 2021; Wright et al., 2021; Ray et al., 2023).

#### 4.2.2 Adverse reactions of inclisiran and safety comparison with siRNA drugs

One of the concerns about siRNA drugs is their possible off-target effects and immune responses (Meng and Lu, 2017; Hu et al., 2020). However, Inclisiran has been developed to reduce these risks by using a highly specific and stable siRNA sequence and a GalNAc conjugate that enhances the delivery to liver cells (Hardy et al., 2021). Inclisiran has a good overall safety profile (Wright et al., 2020). However, recently, there have been some reports that Inclisiran may have adverse reactions such as diarrhea and headache, which has aroused concern about its long-term safety. Further studies could continue monitoring its long-term safety across diverse populations.

### 4.3 Comparison with PCSK9 monoclonal antibody therapy

Compared with PCSK9 monoclonal antibodies, Inclisiran acts downstream by degrading mRNA to achieve sustained suppression of PCSK9, whereas monoclonal antibodies directly block the binding of PCSK9 to LDL receptors (Leiter et al., 2019). The dosing frequency of Inclisiran is once or twice yearly, which is significantly less frequent than the 2-week or monthly injections required for monoclonal antibodies (Ray et al., 2020). This may greatly improve patient compliance. In addition, Inclisiran provides more durable lipid-lowering effects, with a single dose maintaining efficacy for 3–6 months. Overall, as an siRNA therapy, Inclisiran has unique advantages in mechanism, longer duration of action, and more convenient administration compared to monoclonal antibodies. Compared with PCSK9 monoclonal antibodies, Inclisiran demonstrated more durable lipid-lowering effects, which was confirmed by the long-term results of ORION-3 (Ray et al., 2023). The treatment course of PCSK9 monoclonal antibodies is generally 1-2 years, while Inclisiran can maintain the effect for up to 4 years. This may make Inclisiran more suitable for long-term use.

### 4.4 The applications of inclisiran in China nowadays

In addition to conducting clinical trials and obtaining approval in developed countries such as Europe and America, Inclisiran has also initiated a series of investigations and applications in China. However, there are still concerns about the cost and affordability and accessibility of Inclisiran. Inclisiran is priced high, costing about 20,000 yuan per injection, and about 40,000 yuan per year. The drug has not been included in the medical insurance list yet, so patients have to pay for it themselves. The high price of Inclisiran may limit its widespread use in China, which requires improving its accessibility by controlling drug costs and negotiating with medical insurance. We look forward to the real-world application data in the future, to evaluate the cost-effectiveness of Inclisiran in China.

#### 4.4.1 Inclisiran’s benefits for Chinese patients

In the near future, this drug will be more widely available in more cities and hospitals, which will have a positive effect on the quality of life of the Chinese people. Inclisiran is expected to benefit millions of Chinese patients with hypercholesterolemia or mixed dyslipidemia who are not adequately controlled by current therapies (Luo et al., 2023).

#### 4.4.2 Inclisiran’s entry into China

**FIGURE 2:**
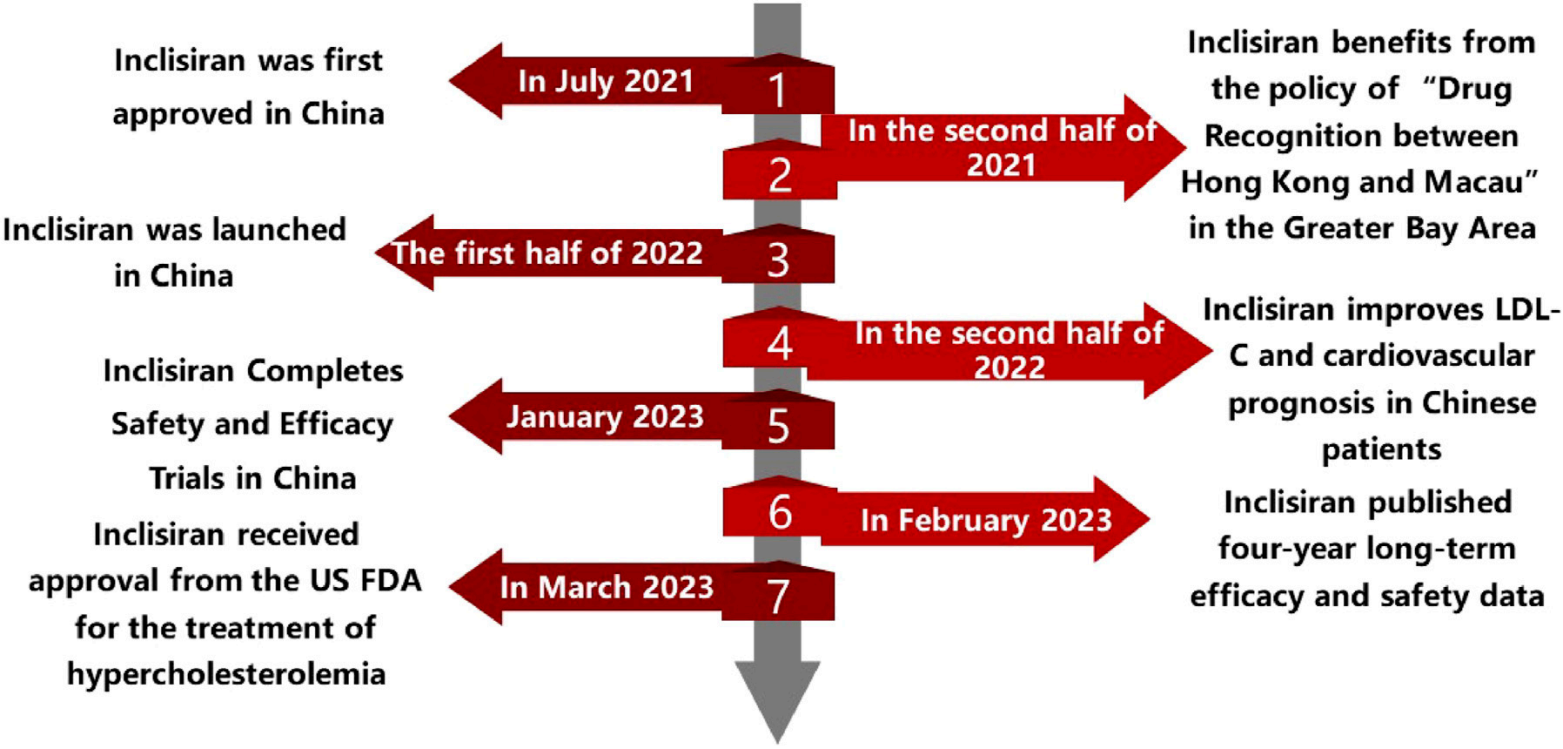
The development and use of Inclisiran in China: a chronological overview.

On July 2 2021, Novartis Pharmaceuticals held a press conference to announce that its new siRNA lipid-lowering drug, Inclisiran, had completed the first injection in China in Boao Super Hospital. The drug was subsequently used in some private hospitals in the Greater Bay Area as the 17th drug approved through the policy of “drug communication between Hong Kong and Macao”. This policy allows the use of drugs that have been approved by the regulatory authorities of Hong Kong or Macao in designated medical institutions in Hainan province without additional approval from the mainland authorities. In this way, the policy can facilitate the access of innovative drugs to Chinese patients and promote the development of the healthcare industry in Hainan. The following timeline (Figure 2) summarizes the main events and dates of the introduction and utilization of Inclisiran in China.

## 5 Discussion

In this review, Inclisiran had been introduced as a novel siRNA lipid-lowering agent that can silence PCSK9 gene expression and lower LDL-C levels with only two or three injections per year. The characteristics and prospects of Inclisiran in terms of its clinical trials, safety and applications in China had also been summarized. In this section, some of the remaining issues and challenges for Inclisiran had been discussed, such as its impact on the risk of type 2 diabetes, its long-term safety and efficacy, its cost-effectiveness and accessibility, and its interactions and synergies with other drugs.

One of the issues that need to be further investigated is the impact of Inclisiran on the risk of type 2 diabetes. Up-regulating LDL receptors in order to reduce LDL-C levels had been shown to have mixed results in terms of its effect on type 2 diabetes incidence. Up-regulating LDL receptors in order to reduce LDL-C levels has been shown to have mixed results in terms of its effect on type 2 diabetes incidence (Ray et al., 2023). Type 2 diabetes is a metabolic disorder that can increase the risk of cardiovascular complications. Some studies suggest that it has a beneficial effect, while others find no significant effect. It is possible that the discrepancy between studies may be due to gender differences, as some studies have reported that women may have a higher risk of developing type 2 diabetes after LDL receptor upregulation than men (Shi et al., 2020). Mendelian randomization had previously proved that the reduction of LDL-C due to genetic variation at PCSK9 does increase the risk of type 2 diabetes (Schmidt et al., 2017). At present, the exact mechanism by which PCSK9 inhibitors increase the risk of type 2 diabetes is unclear. In the same way, it remained to be further discussed whether Inclisiran increased the prevalence of type 2 diabetes.

We believed that the impact of Inclisiran on the risk of type 2 diabetes is an unresolved issue, which may be influenced by gender, genotype and other drugs, and requires more research on individualized, precision and combination therapy to elucidate its mechanism and optimize its application.

Another issue that needs to be addressed is the long-term safety and efficacy of Inclisiran. The results of the clinical trial studies published so far confirmed the efficacy of Inclisiran in lowering LDL-C. However, even with lipid-lowering therapy, there are still some considerable residual risks of cardiovascular disease. These risks are considered to be caused by dysglycemia, hypertension, procoagulant state and inflammation. These are factors that can impair the endothelial function and promote the development of atherosclerosis. Clinical findings regarding lipids have emerged in recent years, and as a result, consensus lipid management guidelines have proposed lower LDL-C control goals. These guidelines are based on the evidence that lower LDL-C levels can reduce the risk of cardiovascular events and mortality in patients with dyslipidemia. Lipid management was gradually changing from “Lower is Better” to “Lower for Longer.” This means that achieving and maintaining low LDL-C levels for a long period of time is more important than short-term fluctuations (Ray et al., 2023). We believed that the long-term safety and effectiveness of Inclisiran is an important and uncertain issue, and more follow-up and observation are needed to assess its impact on the prevention and treatment of cardiovascular disease.

A third issue that needs to be considered is the cost-effectiveness and accessibility of Inclisiran. However, there were still some challenges and limitations for Inclisiran and other siRNA drugs. These drugs can effectively lower LDL-C levels by inhibiting the protein that degrades LDL receptors in the liver. The low frequency of treatment of Inclisiran could improve patients’ compliance and significantly reduce the complication incidence, which may provide a future solution required for LDL-C-lowering treatment. For instance, the long-term safety and efficacy of Inclisiran need to be further evaluated in larger and more diverse populations, especially in patients with high cardiovascular risk or familial hypercholesterolemia. These are patients who may have more severe or resistant forms of dyslipidemia and may require more intensive or novel treatments. Moreover, the cost-effectiveness and accessibility of Inclisiran need to be improved to make it more affordable and available for patients who need it. The current price and supply of Inclisiran might limit its widespread use and adoption in clinical practice (Desai et al., 2022).

A final issue that needs to be explored is the potential interactions and synergies between Inclisiran and other lipid-lowering agents or cardiovascular drugs. There may be additional or synergistic effects of combining Inclisiran with other drugs that can lower LDL-C levels by different mechanisms or that can modulate other cardiovascular risk factors. The cost-effectiveness and accessibility of Inclisiran is an issue to consider, but Inclisiran and other siRNA drugs still face some challenges and limitations that need to be overcome or addressed.

Additionally, the molecular mechanisms and pathways involved in Inclisiran-mediated LDL-C reduction and cardiovascular protection need to be elucidated to reveal the underlying biology and pharmacology of siRNA drugs. This might help to understand the mode of action and the potential off-target effects of Inclisiran and other siRNA drugs. Finally, the development of novel siRNA drugs targeting other genes or molecules related to lipid metabolism or cardiovascular disease might offer new opportunities and possibilities for personalized and precision medicine. These drugs may provide more specific and tailored treatments for different subtypes or phenotypes of patients with dyslipidemia or cardiovascular disease (Scicchitano et al., 2021; Mercep et al., 2022).

## 6 Conclusion

**FIGURE 3:**
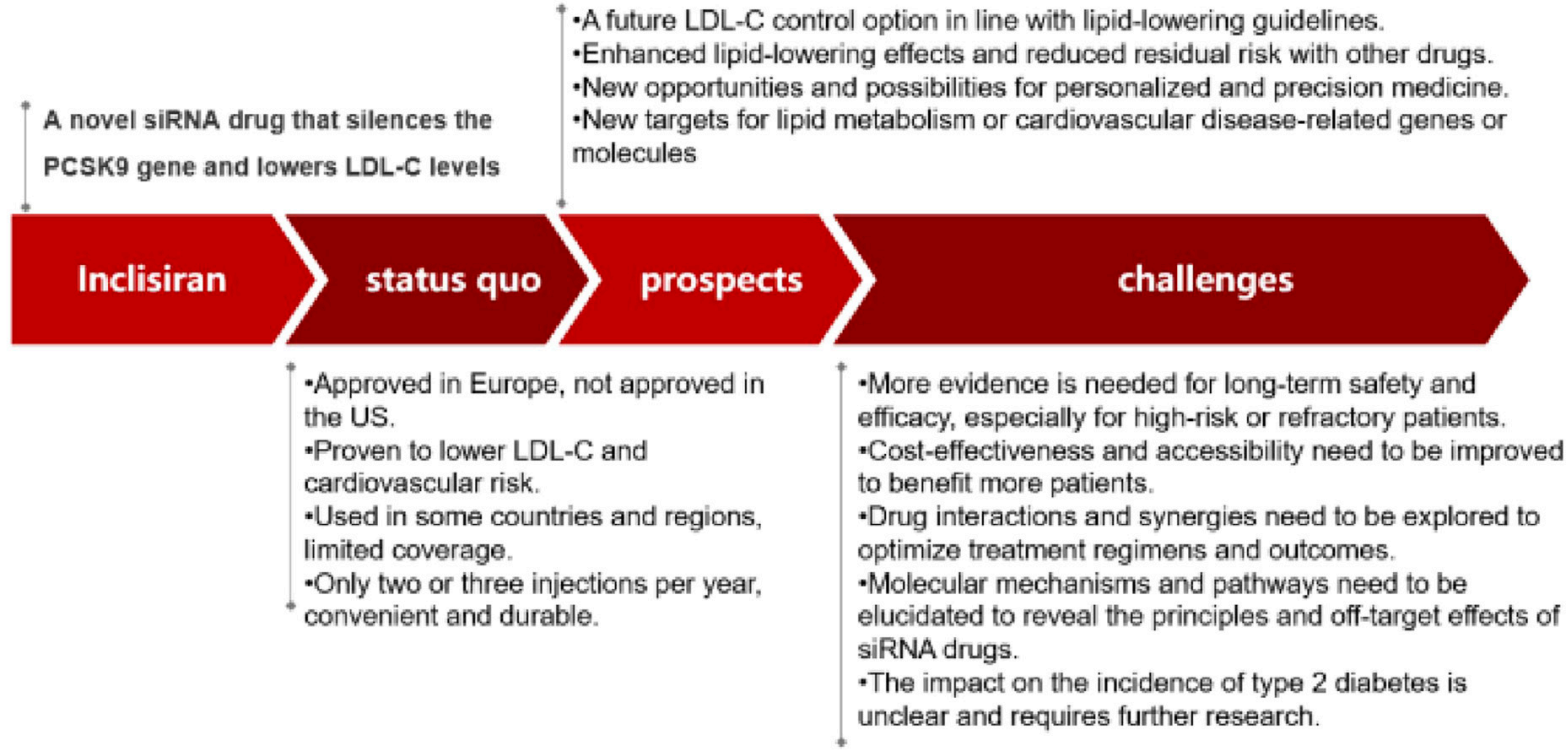
Status quo, prospects and challenges of Inclisiran.

In conclusion, Inclisiran was a promising siRNA lipid-lowering agent that can silence PCSK9 gene expression and lower LDL-C levels with only two or three injections per year. It had shown favorable results in various clinical trials for different types of hypercholesterolemia and cardiovascular disease patients. It had also demonstrated a good safety profile and a potential application in China. Globally, Inclisiran has been approved by the US FDA and the European EMA for the treatment of primary hypercholesterolemia or mixed dyslipidemia. In China, Inclisiran has been approved for the general evaluation of drugs in Hainan Province, and some private hospitals can use this innovative lipid-regulating drug. It is expected that Inclisiran will obtain the approval of China NDAs in the near future. However, there were still some issues and challenges that need to be addressed for Inclisiran, such as its impact on the risk of type 2 diabetes, its long-term safety and efficacy, its cost-effectiveness and accessibility, and its interactions and synergies with other drugs. Moreover, the molecular mechanisms and pathways involved in Inclisiran-mediated LDL-C reduction and cardiovascular protection need to be elucidated, and the development of novel siRNA drugs targeting other genes or molecules related to lipid metabolism or cardiovascular disease may offer new opportunities and possibilities for personalized and precision medicine. Compared with PCSK9 antibodies, Inclisiran had the advantages of less frequent administration, lower injection volume, and potentially lower cost. However, it should be noted that Inclisiran had not yet proven its ability to reduce cardiovascular events and mortality in large-scale trials, while PCSK9 antibodies had already demonstrated such benefits in several studies. Therefore, the clinical outcomes of Inclisiran need to be further confirmed by ongoing or future trials. To summarize the main points of our review, we had provided a graphical abstract in Figure 3 (See Figure 3).

## 7 Prospects

Recently, the indication of Inclisiran has been expanded to populations with high cardiovascular risk factors but without a history of cardiovascular events, for primary prevention. This expanded indication provides a new option for early intervention in high-risk populations. The sustained lipid-lowering effect and convenient dosing regimen of Inclisiran may help improve medication adherence and therapeutic outcomes in these patients. This opens up a promising primary prevention market for Inclisiran. We look forward to more clinical study results to verify the long-term safety and efficacy of Inclisiran in diverse populations.
